# Performance Improvement of Total Ionization Dose Radiation Sensor Devices Using Fluorine-Treated MOHOS

**DOI:** 10.3390/s16040450

**Published:** 2016-03-29

**Authors:** Wen-Ching Hsieh, Hao-Tien Daniel Lee, Fuh-Cheng Jong, Shich-Chuan Wu

**Affiliations:** 1 Minghsin University of Science and Technology, Xinfeng 30401, Taiwan; 2 Treasure Giant Technology Inc., Hsinchu City 30068, Taiwan; danieleewww@gmail.com; 3 Southern Taiwan University of Science and Technology, Tainan 71005, Taiwan; fcjong@totalbb.net.tw; 4 National Nano Device Laboratories, Hsinchu 30078, Taiwan; scwu@narlabs.org.tw

**Keywords:** SONOS, NVM, sensor, gamma ray

## Abstract

Fluorine-treated titanium nitride–silicon oxide–hafnium oxide–silicon oxide–silicon devices (hereafter F-MOHOS) are candidates for total ionization dose (TID) radiation sensor applications. The main subject of the study reportedherein is the performance improvement in terms of TID radiation-induced charge generation effect and charge-retention reliability characterization for F-MOHOS devices. In the case of F-MOHOS TID radiation sensors, the gamma radiation induces a significant decrease of threshold voltage *V_T_* and the radiation-induced charge density is nearly six times larger than that of standard metal–oxide–nitride–oxide–silicon MONOS devices. The decrease of *V_T_* for F-MOHOS after gamma irradiation has a strong correlation to the TID up to 5 Mrad gamma irradiation as well. The improvement of charge retention loss for F-MOHOS devices is nearly 15% better than that of metal–oxide–hafnium oxide–oxide–silicon MOHOS devices. The F-MOHOS device described in this study demonstrates better feasibility for non-volatile TID radiation sensing in the future.

## 1. Introduction

The total ionizing dose (TID) radiation-induced charging effect is a major application concern for the operation of electronic devices in advanced X-ray lithography semiconductor manufacturing processes and outer space applications. When a metal-silicon dioxide-silicon (MOS) structure is irradiated by gamma rays, positive charges build-up at the Si-SiO_2_ interface and an interface state occurrs in the structure [1]. The radiation-induced charging effects of a metal–nitride–oxide–silicon (MNOS) device with stacked insulation layers composed of silicon nitride and silicon dioxide have been reported [2]. The radiation-induced charging effects on traditional silicon–oxide–nitride–oxide–silicon (SONOS) nonvolatile memory (NVM) devices have also been studied before. [3,4]. Until now, little was known about the radiation response of SONOS–like devices with high k charge-trapping structure [4,5]. High-k gate dielectrics have been used for reducing transistor gate leakage current in advanced nano-scale CMOS device technology [5]. Recently, conventional SONOS flash memory was replaced with silicon–oxide–hafnium oxide–oxide–silicon SOHOS devices (hafnium-based SONOS-like devices with high k material as charge-trapping structure). However, SOHOS devices have worse data retention characteristics, as is well known [5]. The effects of radiation response on a few SOHOS-like devices have been reported [4,5], but the charge retention reliability of the SOHOS device as TID radiation sensor has not been well studied and it will be the main subject of this study. In order to improve the radiation-induced charge density and charge retention reliability of SOHOS device for non-volatile TID radiation sensor applications, a titanium nitride–silicon oxide–hafnium oxide–silicon oxide–silicon device with CF_4_ plasma treated hafnium oxide HfO_2_ (hereafter F-MOHOS) was fabricated. The electrical performance of F-MOHOS, including radiation-induced charge generation effect and charge retention reliability characterization, are the main subjects of discussion in this paper, which reports a study of different types of F_-_treated MOHOS to manipulate the radiation-induced charging effects and charge retention reliability characterization of F_-_treated HfO_2_. In contrast to the previous publication [4], the MOHOS devices were irradiated by gamma irradiation with negative gate bias stress (NVS). The NVS application increases the survival yield of radiation-induced electron-hole pairs from the initial recombination process and also increases the radiation-induced charging yield of the MOS type devices [6].

## 2. Experimental Section

The MOHOS devices prepared with various F_-_treated HfO_2_ materials are listed in Table 1. MOHOS structures were fabricated on p-type resistivity 15–25 Ω-cm Si <100> substrate. To fabricate MONOS devices, we used thermal silicon oxide SiO_2_ as tunneling oxide, CVD silicon nitride Si_3_N_4_ for the trapping layer, and CVD TEOS SiO_2_ as blocking oxide. The tunneling oxide (SiO_2_) was formed on the wafers by using an advanced clustered vertical furnace. After the tunneling oxide formation, silicon nitride (hereafter, nitride, Si_3_N_4_) was deposited as the charge-trapping layer by low-pressure chemical vapor deposition (LPCVD) for MONOS devices.

**Figure 1:**
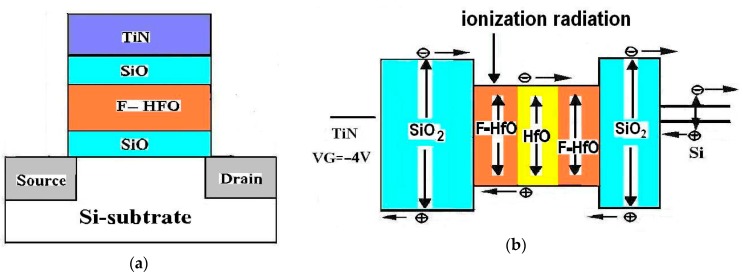
(**a**) Cross-section view of F-MOHOS devices; (**b**) Charges generation and trapping states in the FAB-MOHOS device after gamma irradiation.

For MOHOS devices, HfO_2_ films (10~20 nm) were deposited as the charge-trapping layers, with Hf(*tert*-butoxy)_2_(mmp)_2_ precursor in a metal organic chemical vapor deposition (MOCVD) system at 400 ~ 550 °C. For F-MOHOS devices, CF_4_ plasma treatment with 30 sccm at 50 W for 30 s was performed on MOHOS. To manipulate the radiation-induced charging effects in F_-_treated HfO_2_, three type of F_-_treated MOHOS were prepared: (1) “FB” type MOHOS (hereafter FB-MOHOS), CF_4_ plasma treatment before HfO_2_ deposition; (2) “FA” type MOHOS (hereafter FA-MOHOS), CF_4_ plasma treatment after HfO_2_ deposition; (3) “FAB” type MOHOS (hereafter FAB-MOHOS), CF_4_ plasma treatment both before and after HfO_2_ deposition. The SiO_2_–Si_3_N_4_–SiO_2_ (hereafter ONO) gate stack consists of a 100 Å–200 Å silicon nitride and 50 Å–150 Å bottom and top silicon oxides. TiN metal gate (200–400 nm) was formed by DC sputtering for the control gate. After gate patterning, source and drain were formed by implantation with arsenic atoms which were activated at 900 °C for 30 s. Figure 1a shows a cross-section view of the MOHOS devices. For comparison, all the devices listed in Table 1 have the same tunneling oxide, charge-trapping layer and blocking oxide layer thickness. A MOHOS device with dimensions W x L = 0.1 × 0.1 mm^2^ was used in this paper.

For gamma TID data writing, in this study all the devices listed in Table 1 were exposed to ^60^Co gamma radiation with gate negative bias stress (NVS, *V_G_* = −4 V). For the gamma TID data read, *V_T_* shifting was measured at room temperature using a HP4156A parameter analyzer. The of I_D_ − V_G_ curve experimental results of the MOHOS device pre-irradiation and post-irradiation were compared by a computer-controlled HP4156A parameter analyzer at room temperature. Figure 1b shows the charge generation and trapping states of the gate dielectric in the FAB-MOHOS device after gamma irradiation.

## 3. Results and Discussion

### 3.1. Radiation-Induced Charging Effect of F-MOHOS after Gamma Irradiation

**Figure 2:**
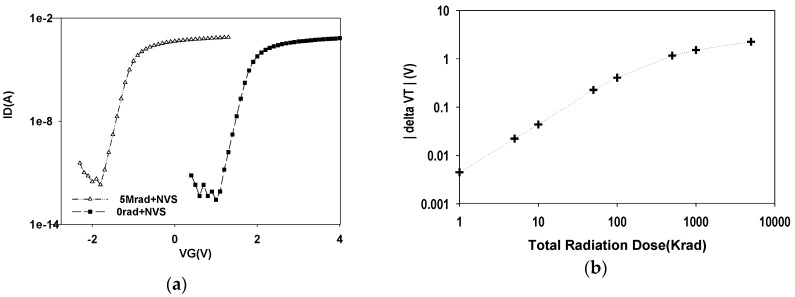
(**a**) The *I_D_ − V_G_* curve for MOHOS device before and after 5 Mrad TID gamma irradiation; (**b**) The |delta *V_T_*| increase as a function of gamma irradiation TID for MOHOS device.

As illustrated in Figure 2a, the *I_D_ − V_G_* curve of MOHOS was shifted to the left after 5 Mrad TID of gamma irradiation. This implies that gamma irradiation induces a decrease of *V_T_* for MOHOS. The amount of decrease of *V_T_* is about 2.9 V. It is considered that the change is due to an increase in the net positive trapped charges in the HfO_2_ charge-trapping layer after gamma irradiation. The negative *V_T_* shift result agrees with those of previous studies [3,4]. These radiation-induced shifts in the irradiated device are a combination of two effects; the first effect is a result from the loss of stored negative charge in the HfO_2_ trapping layer and the second effect is due to a build-up of positive charge resulted from asymmetric trapping of electrons and holes in the HfO_2_ trapping layer.

The |*delta V_T_*| of the MOHOS device increases as a function of gamma TID, as indicated in Figure 2b. It also shows a quasi-linear correlation of |*delta V_T_* | *vs.* gamma TID below 100 krad in log scale, but |*delta V_T_*| increases more sharply after gamma irradiation at levels up to 100 krad TID. This result is in agreement with those of previous studies [4].

**Figure 3:**
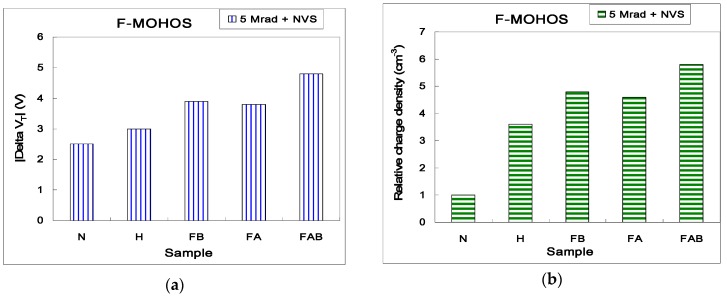
(**a**) |Delta V_T_| for various F-MOHOS devices after 5 Mrad TID irradiation; (**b**) Relative charge density for various F-MOHOS devices after 5 Mrad TID irradiation.

The radiation-induced |*delta V_T_*| and charge density comparisons after 5 Mrad TID gamma irradiation for various F-MOHOS devices shown in Table 1 are illustrated in Figure 3a,b The trapped charge density can be calculated by the Terman method [5]. As shown in Figure 3a, the radiation-induced *V_T_* shift of MOHOS is more significant than that of MONOS, which results from more radiation-induced charges in the HfO_2_ trapping layer than in the Si_3_N_4_ charging layer. In addition, the F-MOHOS devices with various F treatments (FA-, FB- and FAB-MOHOS) all demonstrate higher degrees of *V_T_* shift and higher radiation-induced charge density than the MOHOS devices. These results are contributed by a higher radiation-induced charging effect on these F-MOHOS devices than that on MOHOS devices. Note that the radiation-induced charge density of the FAB-MOHOS device is six times larger than that of traditional MONOS devices. The FAB-MOHOS device with larger F-treatment volume in HfO_2_ has the higher radiation-induced charge density than the FA-MOHOS and FB-MOHOS devices after gamma irradiation.

### 3.2. V_T_ Stability vs. Retention Time

**Figure 4:**
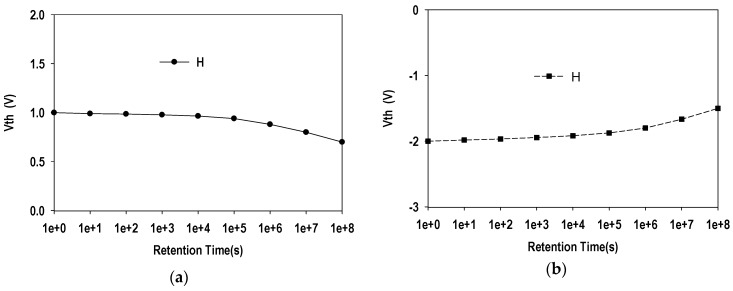
The *V_T_ vs.* retention time for MOHOS device: (**a**) before gamma irradiation; (**b**) after 5 Mrad gamma irradiation.

In this section, the radiation-induced charges-retention reliability characteristics of the F-MOHOS devices are discussed and these are the important electrical properties that need to be verified for their potential application in TID radiation sensors in this study. The *V_T_* stability *vs.* time for MOHOS under *V_G_* = −4 V before gamma irradiation and after 5 Mrad gamma irradiation is illustrated in Figure 4a,b respectively.

It is noted that the decrease of the *V_T_* with time for the pre-irradiated MOHOS device is a result of stored negative-charge tunneling out from the HfO_2_ trapping layer. Note that the increase of the *V_T_* with time for the post-irradiated MOHOS device is a result of radiation-induced positive charges tunneling out from the HfO_2_ trapping layer.

**Figure 5:**
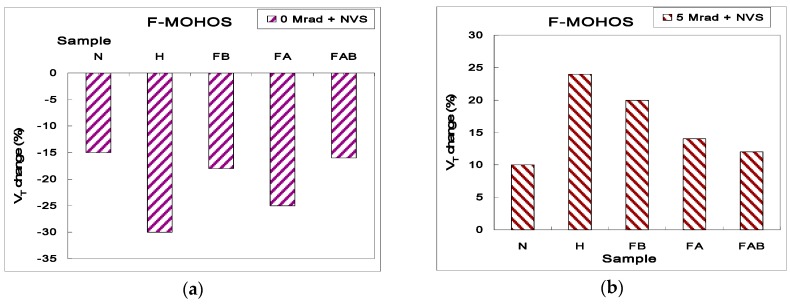
The *V_T_* change with 10-years retention time for various F-MOHOS devices under *V_G_* = −4 V after (**a**) 0 Mrad gamma irradiation; (**b**) 5 Mrad gamma irradiation.

Figure 5a shows the *V_T_* stability *versus* time with NVS (*V_G_* = −4 V) for various F-MOHOS devices shown in Table 1 before gamma irradiation. It is seen that the device with HfO_2_ as the charge-storage layer shows the worst charge retention reliability characteristics compared with Si_3_N_4_. The worse charge storage capacity in the MOHOS device may be attributed to tunneling leakage current induced by interface trap states [7]. As shown in Figure 5a, the F-MOHOS devices demonstrate better charge-retention reliability characteristics than MOHOS ones before gamma irradiation, which is because deep negative-charge traps in F treated trapping HfO_2_ lead to less negative-charge loss and a better negative charge-retention reliability characteristics for the pre-irradiated F-MOHOS than the pre-irradiated MOHOS [7]. However, the FB-MOHOS device has better charge-retention reliability characteristics than the FA-MOHOS devices before gamma irradiation. Because the probability of stored negative-charge tunneling out from bottom of trapping HfO_2_ to tunneling oxide is higher (compared to that from top of trapping HfO_2_ to blocking oxide) for the pre-irradiated F-MOHOS device under NVS. Therefore, the FB-MOHOS device with deeper negative-charge traps at the bottom of HfO_2_ shows better charge-retention reliability characteristic than the FA-MOHOS devices before gamma irradiation.

Figure 5b shows the *V_T_* stability *vs.* time under *V_G_* = −4 V for various F-MOHOS devices after 5 Mrad TID gamma irradiation. We note that the FA-MOHOS demonstrate worse charge-retention reliability characteristics than the FB-MOHOS after 5 Mrad gamma irradiation because the probability of radiation-induced positive charges tunnel-out from the top of trapping nitride to blocking oxide is higher (compared to that from bottom of trapping nitride to tunneling oxide) for the 5 Mrad gamma irradiated F-MOHOS device under NVS. Therefore, the FA-MOHOS device with more deep negative-charge traps at the top of HfO_2_ shows better charge-retention reliability characteristic than the FB-MOHOS devices after 5 Mrad gamma irradiation. Furthermore, the F treatment process during HfO_2_ deposition should be considered for the traded-off between pre-irradiated and post irradiated charge-retention reliability. Therefore, the FAB-MOHOS device with deeper negative-charge traps both at the top and bottom of HfO_2_ is suggested for improvement of charge retention reliability characteristic both before gamma irradiation and after 5 Mrad gamma irradiation.

## 4. Conclusions

As shown by the experimental data, F treatment during HfO_2_ deposition is a very effective process for enhancing the radiation-induced charging effect of MOHOS devices. It can be explained by the fact that the enhanced radiation-induced charging effect of F-MOHOS was induced by more radiation-induced positive charges in the F-treated HfO_2_ trapping layer. In addition, the F treatment process during HfO_2_ deposition should be considered for the trade-off between pre-irradiated and post-irradiated charge-retention reliability. Therefore, the FAB-MOHOS device is suggested for improvement of charge retention reliability characteristics both before gamma irradiation and after 5 Mrad gamma irradiation. The results show that F-MOHOS devices with F-treated HfO_2_ charge-trapping layers can be potential candidate nonvolatile TID radiation sensors in the future.

## Figures and Tables

**Table 1:** MOHOS devices prepared with various F treated HfO_2_ as charge-trapping layer.

Split	N	H	FB	FA	FAB
Charge-trapping layer	Si_3_N_4_	HfO_2_	HfO_2_	HfO_2_	HfO_2_
F treatment	no	no	Before HfO_2_ deposition	After HfO_2_ deposition	before and after HfO_2_ deposition
